# Evaluation of the clinical practice guidelines and consensuses on calcium and vitamin D supplementation in healthy children using the Appraisal of Guidelines for Research and Evaluation II instrument and Reporting Items for Practice Guidelines in Healthcare statement

**DOI:** 10.3389/fnut.2022.984423

**Published:** 2022-09-27

**Authors:** Lanzhi He, Pengxiang Zhou, Xin Zhou, Shuxia Tian, Jing Han, Suodi Zhai

**Affiliations:** ^1^Department of Pharmacy, Peking University Third Hospital, Beijing, China; ^2^Department of Pharmacy, Jiangbin Hospital of Guangxi Zhuang Autonomous Region, Nanning, China; ^3^Institute for Drug Evaluation, Peking University Health Science Center, Beijing, China; ^4^Department of Pharmacy, Children’s Hospital Affiliated to Capital Institute of Pediatrics, Beijing, China; ^5^Department of Pharmacy, Tianjin Nankai Hospital, Tianjin, China; ^6^Department of Internal Medicine, Affiliated Cancer Hospital of Zhengzhou University, Zhengzhou, China

**Keywords:** healthy children, calcium supplementation, vitamin D, guidelines, quality assessment

## Abstract

**Background:**

This study aimed to assess the methodological and reporting quality of the guidelines and consensus on calcium and vitamin D supplementation in healthy children, and the consistency of these recommendations.

**Methods:**

A systematic search of relevant guideline websites and databases, including PubMed, Embase, CNKI, WangFang, and SinoMed, was undertaken from inception to April 7, 2021, by two independent reviewers who assessed the eligible guidelines using the validated Appraisal of Guidelines for Research and Evaluation Instrument II (AGREE II) and the Reporting Items for Practice Guidelines in Healthcare (RIGHT) tools. Overall, the between-reviewer agreement was evaluated using an intra-class correlation coefficient.

**Results:**

A total of 24 guidelines and consensuses from 2002 to 2022 were identified from China, the United States, Canada, France, Australia, New Zealand, Europe, and other countries and regions. These were of mixed quality, and scored poorly in the rigor of development, editorial independence, and applicability of the domains of AGREE II. Among the seven domains of the RIGHT checklist, domain one (basic information) had the highest reporting rate (69.3%), whereas domain five (review and quality assurance) had the lowest reporting rate (11.5%). The overall quality of the included guidelines and consensuses was low. Only 12 guidelines were recommended, with modifications. The recommended calcium intake for children of different ages varies greatly (400–1,150 mg/day). Among the included guidelines and consensuses, a vitamin D (VD) prevention dose of 400 IU/day in infants was generally considered safe, and 25-hydroxyvitamin-D [25(OH)D] levels of <20 ng/mL (50 nmol/L) or 20–30 ng/mL (50–75 nmol/L) indicated VD deficiency or insufficiency. However, the recommended amount of VD for children of different age groups and risk strata differed considerably (400–4,000 IU/day or 10–100 μg/day). The choice of VD2 or VD3 supplements and sunlight exposure also differed across the guidelines and consensuses.

**Conclusion:**

There is considerable variability in calcium and VD guidelines and consensus development methods in calcium and VD supplementation for healthy children. Therefore, efforts are necessary to strengthen the methodological rigor of guideline development and utilize the best available evidence to underpin recommendations.

## Introduction

Calcium, the most abundant mineral element in the human body, is pivotal for good bone health and can enhance muscle function and immunity. Vitamin D (VD) plays a critical role in regulating the balance between calcium metabolism and bone formation in the human body. Currently, calcium or VD deficiency is one of the biggest global public health problems, and studies from both developed and developing countries have shown that calcium and vitamin D deficiency is highly prevalent in children (1–3). Current dietary reference values for these nutrients in children (e.g., range between 200 and 1,000 mg for Ca and 400 and 800 IU or 10–20 μg for VD) and that prevalence of VD insufficiency varies also due to variations in cut-offs of 25(OH)D used to define this. However, the prevalence of insufficiency varies considerably across countries and among subpopulations because of differences in risk factors, especially skin pigmentation, sunlight exposure, and dietary VD intake. Optimal calcium and VD status are crucial in the early years of life, as VD deficiency can lead to nutritional rickets, hypocalcemia, and impacts on the normal growth and development of children and peak bone mass (4, 5).

At present, several guidelines are accessible on calcium and VD supplementation for healthy children worldwide. However, the recommendations differ in terms of the necessity of routine calcium and VD supplementation in healthy children, the dosage and upper limit of supplementation, and the source of supplementation, which complicates the clinical decision-making and limiting the choices available to patients and their families for these issues. Clinical practice guidelines are based on systematic reviews of evidence that balance the benefits and harms of different interventions and provide patients with recommendations on the best health practices (6). High-quality practice guidelines are vital for improving clinical diagnoses and treatment behaviors, and for improving medical quality and results (7). Therefore, it is necessary to use tools such as the Appraisal of Guidelines for Research and Evaluation Instrument II (AGREE II) (8) and the Reporting Items for Practice Guidelines in Healthcare (RIGHT) (9) to evaluate the methodology and reporting quality of the guidelines and summarize the current best recommendations, combined with the best current evidence-based evidence to provide a reference for calcium and VD supplementation in healthy children.

In this study, we aimed to evaluate the characteristics, methodological and reporting quality, and related recommendations of clinical practice guidelines and consensuses on calcium and VD supplementation in healthy children.

## Materials and methods

### Review protocol

This study was performed in accordance with the guidelines from the preferred reporting items for systematic reviews and meta-analyses (PRISMA).

### Search strategy

PubMed, Embase, CNKI, WangFang Data, and SinoMed databases, and the official websites of AHRQ,^1^ GIN,^2^ NICE,^3^ SIGN,^4^ IPGRP,^5^ WHO,^6^ AAP,^7^ ASN,^8^ and FENS^9^ were systematically searched. The search strategy combined the keywords (“calcium” or “vitamin D”) with various expanded keywords (“guideline” or “consensus” or “recommendation”) from inception to April 7, 2021 (last update search May 6, 2022). A supplementary search was also performed in the references of the included studies. The details of the search strategy are shown in Supplementary material 1.

### Inclusion and exclusion criteria

The inclusion criteria were as follows: (1) papers that included guidelines or consensus on calcium or VD supplementation (including calcium-containing dairy products) for healthy children (age < 18 years; we discerned “healthy children” according to the recommendations of the text and extracted concepts such as adequate intake (AI) and reference nutrient intake (RNI); while relevant statements of “non-healthy children”, such as premature infants, low birth weight infants, overweight/obese children, nutritional rickets, and the use of drugs affecting vitamin D metabolism, were not included in the consideration of this study); (2) papers in which the guideline developers were professionals or had academic associations in healthcare; and (3) papers in which the language was limited to English and Chinese.

The exclusion criteria were as follows: (1) papers in which the target population was preterm infants; (2) papers in which the specific recommended dose was not mentioned; (3) papers with an unavailable full text; (4) papers that used older versions of the included guidelines; and (5) papers that included guidelines reviews, translations, interpretations, or other non-original guidelines.

### Literature screening and data extraction

According to the inclusion and exclusion criteria, two reviewers (H.L.Z. and Z.P.X.) independently extracted the characteristics of the included guidelines and consensuses based on a standardized and pre-designed form. The extracted items included the first author, publication year, country or region, developing organization, guideline development methods, assessment methods of evidence quality, and recommendation formation methods. Any disagreement was discussed or resolved by consulting a third reviewer (Z.S.D.).

### Methodological and reporting quality of the included guidelines and consensuses

Before the formal AGREE II and RIGHT evaluations, two reviewers (H.L.Z. and Z.X.) systematically learned the AGREE II online tutorial training^10^ and relevant examples, only when two reviewers have high consistency (ICC > 0.85), the formal appraisal was conducted.

Intra-class correlation coefficients (ICC) were used to assess the consistency of the reviewers’ understanding of each item. Then, the guidelines and consensuses were evaluated independently by the two reviewers in line with the AGREE II and RIGHT statements, and any disagreement was resolved by discussion with a third reviewer (Z.P.X.).

Appraisal of Guidelines for Research and Evaluation Instrument II (8) was used to evaluate the methodological quality of the guidelines and consensuses. It consists of 23 key items organized within six domains. Each item was scored from one (strongly disagree) to seven (strongly agree). The scores were graded according to the completeness and quality of reporting. The total score for each domain was obtained by summing the scores of both individual reviewers for all items in a certain domain and standardized as follows: (obtained score – minimal possible score)/(maximal possible score – minimal possible score). The overall AGREE II assessment (recommend, recommend with modifications, or do not recommend) was also independently determined by each reviewer.

The RIGHT checklist provided reviewers with clear, explicit descriptions of guideline development processes and procedures and the evidence used to formulate each recommendation. It consists of 22 key items organized within seven domains. Each item was scored as fully reported (1 point), partially reported (0.5 points), not reported (0 points), or not applicable (0 points). The reporting rate was defined as the ratio of the obtained score to the maximum possible score.

### Analysis of recommendations

We comprehensively summarized the recommendations of calcium and VD supplementation guidelines or consensuses in healthy children and compared their similarities and differences. We discussed the recommended intake and supplementation of calcium and VD for healthy children of all ages; the appropriate level of 25-hydroxyvitamin-D (25(OH)D), VD2, or VD3 supplements; and sunlight exposure suggestions.

### Statistical analysis

Microsoft Excel 2019 was used to sort out the scaled scores of AGREE II and RIGHT in each domain. Descriptive statistics, including median and interquartile range (IQR) (or mean and standard deviation [SD]) and percentage analyses, were performed for the standardized scores of each domain. The consistency between the two reviewers was measured by calculating the ICC with a 95% confidence interval (CI) using IBM SPSS 25.0. ICC scores of >0.75 indicated acceptable consistency (10).

## Results

**FIGURE 1:**
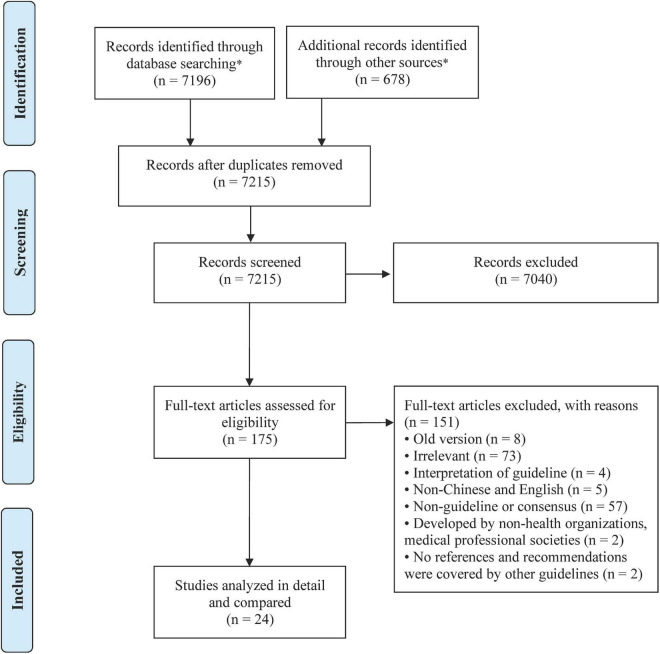
Preferred reporting items for systematic reviews and meta-analyses (PRISMA) diagram for the included studies. *The specific databases searched and the number of literatures detected are as follows: PubMed (*n* = 549), Embase (*n* = 4,371), CNKI (*n* = 1,019), WangFang (*n* = 766), SinoMed (*n* = 491), AHRQ (*n* = 79), GIN (*n* = 16), NICE (*n* = 245), SIGN (*n* = 0), IPGRP (*n* = 19), WHO (*n* = 11), AAP (*n* = 146), ASN (*n* = 50), FENS (*n* = 107), hand search for relevant references (*n* = 5).

The search initially yielded 7,874 records, and 24 guidelines and consensuses were finally included, 12 guidelines were evidence-based, four were non-evidence-based, and eight were consensus, all of which were developed by local or international medical societies. One guideline (11) was globally developed, and the others were developed in 12 different countries and regions, including China (4, 5, 12, 13), the USA (14–16), Europe (17–19), India (20), Australia and New Zealand (21, 22), the United Arab Emirates (23), France (24, 25), Canada (26), Poland (27), Italy (28), Germany (29, 30), and Latin America (31). Figure 1 shows the flow diagram of the study selection process. Studies were published (or updated) from 2002 to 2022, of which 12 guidelines were evidence-based guidelines (11, 13, 15, 18, 20–22, 25–28, 31), four were non-evidence-based guidelines (14, 23, 24, 32), and eight were consensus guidelines (4, 5, 12, 16, 17, 19, 29, 30). A summary of the characteristics of the included guidelines and consensuses is presented in Table 1.

**TABLE 1:** Characteristics of the included guidelines and consensus.

Included guidelines	Country/Region	Publishing organization/ Group	Type of guideline	Recommendations for calcium or VD	Evidence grading and recommendation strength tools
Hochberg et al. (19)	Europe	ESPEBC	Consensus	Both	—
Godel (26)	Canada	CPS	Evidence-based guide	VD	CTFPHC
Sanders et al. (21)	Australia and New Zealand	ANZBMS/OA	Evidence-based guide	Calcium	NHMRC (No recommended strength)
Holick et al. (15)	America	ES	Evidence-based guide	VD	GRADE
Vidailhet et al. (24)	France	CNFSP	Non-evidence-based guide	VD	—
GNS (29)	Germany	GNS	Consensus	VD	—
Braegger et al. (18)	Europe	ESPGHAN	Evidence-based guide	VD	—
SAHM (16)	America	SAHM	Consensus	VD	—
Paxton et al. (22)	Australia and New Zealand	ANZBMS/OA	Evidence-based guide	VD	NHMRC (No recommended strength)
GNS (30)	Germany	GNS	Consensus	Calcium	—
Płudowski et al. (32)	Central Europe	Consensus group	Non-evidence-based guide	VD	—
Golden et al. (14)	America	AAP	Non-evidence-based guide	Both	—
Munns et al. (11)	Multi-regional and multi-organization cooperation around the world	Consensus group	Evidence-based guide	Both	GRADE
Grossman et al. (17)	Europe	EAP	Consensus	VD	—
CSOBMR (12)	China	CSOBMR	Consensus	VD	—
Haq et al. (23)	United Arab Emirates	UAE/GCC	Non-evidence-based guide	VD	—
Rusinska et al. (27)	Poland	Consensus group	Evidence-based guide	VD	GRADE
Saggese et al. (28)	Italy	SIP	Evidence-based guide	VD	—
CHSCPMA (4)	China	CHSCPMA	Consensus	Calcium	—
Palacios et al. (31)	Latin America	SIBOMM	Evidence-based guide	VD	—
CHSCPMA (5)	China	CHSCPMA	Consensus	VD	—
Gupta et al. (20)	India	IAP	Evidence-based guide	VD	OCEBM
Bacchetta et al. (25)	France	Consensus group	Evidence-based guide	Both	AAP
PSCMA (13)	China	PSCMA	Evidence-based guide	VD	GRADE

IAP, Indian Academy of Pediatrics; Consensus group: representing 11 international scientific organizations (pediatric endocrinology, pediatrics, nutrition, epidemiology, public health, and health economics); GRADE, Grading of Recommendations Assessment, Development and Evaluation; ANZBMS and OA, Australian and New Zealand Bone and Mineral Society and Osteoporosis Australia; NHMRC, National Health and Medical Research Council; AAP, American Academy of Pediatrics; EAP, European Academy of Pediatrics; UAE and GCC, United Arab Emirates and Gulf Cooperation Council; ES, Endocrine Society; ESPGHAN, European Society for Pediatric Gastroenterology, Hepatology, and Nutrition; SAHM, Society for Adolescent Health and Medicine; CNFSP, Committee on Nutrition of the French Society of Pediatrics; CPS, Canadian Pediatric Society; CTFPHC, Canadian Task Force on Preventive Health Care; Export Committee: national consultants and experts in the field; SIP, Italian Society of Pediatrics; SIBOMM, the Iberoamerican Society of Osteoporosis and Mineral Metabolism; CHSCPMA, Child Health Society of Chinese Preventive Medicine Association; CSOBMR, Chinese Society of Osteoporosis and Bone Mineral Research; GNS, German Nutrition Society; PSCMA, Pediatrics Society of Chinese Medical Association.

### Evaluation of methodological quality (Appraisal of Guidelines for Research and Evaluation II)

Overall, the ICC scores were > 0.80 in each domain, suggesting good agreement between the reviewers. Only 12 (50%) guidelines and consensuses were “recommended with modification,” and the rest were “not recommended.” The quality of the domains was heterogeneous. With respect to the overall domain scores across all guidelines or consensuses, the first domain (scope and purpose) scored the highest with a median score of 83.3%, and the third domain (rigor of development) scored the lowest with a median score of 18.8%. The sixth domain (editorial independence) and fifth domain (applicability) scored < 30%. Regarding the clarity of presentation, 88% (21/24) of the recommendations were specific and unambiguous, 79% (19/24) presented different options for management of the condition or health issues clearly, and 71% (17/24) had easily identifiable key recommendations. In contrast, the weakest area of the analyzed guidelines was rigor development. Only one fifth of the guidelines and consensus were externally reviewed by experts before publication (21%, 5/24), and even fewer guidelines provided a procedure for updating the guidelines (8%, 2/24). A comprehensive evaluation was conducted in combination with various domains, and the overall quality of the included guidelines and consensuses was low. The ICC and AGREE II scores of each domain are summarized in Table 2.

**TABLE 2:** Appraisal of Guidelines for Research and Evaluation II (AGREE II) scaled domain score (%) and ICC score of included guidelines and consensus.

Included guidelines	Scope and Purpose	Stakeholder involvement	Rigor of development	Clarity and presentation	Applicability	Editorial independence	Overall assessment
Hochberg et al. (19)	80.6	44.4	11.5	77.8	25.0	0	N
Godel et al. (26)	86.1	27.8	21.9	69.4	22.9	4.2	RM
Sanders et al. (21)	69.4	50.0	33.3	72.2	29.2	58.3	RM
Holick et al. (15)	91.7	58.3	53.1	94.4	43.8	100.0	RM
Vidailhet et al. (24)	83.3	36.1	13.5	58.3	20.8	0	N
GNS (29)	47.2	11.1	12.5	33.3	10.4	0	N
Braegger et al. (18)	83.3	47.2	22.9	80.6	27.1	100.0	RM
SAHM (16)	66.7	27.8	9.4	66.7	27.1	0	N
Paxton et al. (22)	83.3	50.0	50.0	77.8	41.7	50.0	RM
GNS (30)	66.7	22.2	12.5	86.1	12.5	0	N
Płudowski et al. (32)	58.3	50.0	15.6	86.1	25.0	0	N
Golden et al. (14)	88.9	50.0	12.5	66.7	33.3	100.0	N
Munns et al. (11)	91.7	58.3	50.0	80.6	47.9	83.3	RM
Grossman et al. (17)	86.1	44.4	8.3	52.8	29.2	0	N
CSOBMR (12)	41.7	50.0	10.4	63.9	29.2	0	N
Haq et al. (23)	88.9	47.2	15.6	75.0	45.8	0	N
Rusinska et al. (27)	83.3	50.0	37.5	97.2	25.0	100.0	RM
Saggese et al. (28)	91.7	66.7	44.8	86.1	31.3	100.0	RM
CHSCPMA (4)	55.6	33.3	4.2	36.1	14.6	0	N
Palacios et al. (31)	69.4	22.2	35.4	77.8	35.4	87.5	RM
CHSCPMA (5)	72.2	41.7	9.4	61.1	25.0	0	N
Gupta et al. (20)	88.9	61.1	53.1	94.4	45.8	50.0	RM
Bacchetta et al. (25)	88.9	55.6	74.0	94.4	33.3	91.7	RM
PSCMA (13)	83.3	72.2	69.8	88.9	50.0	91.7	RM
ICC (95%CI)	0.84 (0.76–0.90)	0.98 (0.97–0.99)	0.96 (0.95–0.97)	0.91 (0.86–0.94)	0.89 (0.84–0.93)	0.99 (0.98–0.99)	—
Median score (IQR)	83.3 (21.5)	48.6 (20.2)	18.8 (37.0)	77.8 (21.5)	29.2 (15.1)	27.1 (91.7)	—

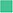
 100%∼80%; 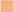 79%∼60%; 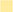 59%∼40%; 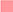 39%∼20%; 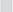 <20%.

ICC, Intra-class Correlation Coefficient; RM, Recommended with modifications; N, Not Recommended; SAHM, Society for Adolescent Health and Medicine; CSOBMR, Chinese Society of Osteoporosis and Bone Mineral Research; CHSCPMA, Child Health Society of Chinese Preventive Medicine Association; GNS, German Nutrition Society; PSCMA, Pediatrics Society of Chinese Medical Association.

### Evaluation of reporting quality (Reporting Items for Practice Guidelines in Healthcare statement)

The ICC scores for all domains were > 0.80, demonstrating considerable consistency between the reviewers. The mean overall reporting rate was 42.3 ± 14.3%, ranging from 24.3 to 69.7%. Among the seven domains, basic information received the highest reporting rate (69.3%), whereas review and quality assurance received the lowest reporting rate (11.5%). A comprehensive evaluation of various domains revealed that only seven guidelines had an overall score of over 50% (11, 13, 15, 20, 25, 27, 28). Most were well-identified in the titles (83%, 20/24). In a majority of guidelines, the recommendations were clear, explicit, and implementable (71%, 17/24), and abbreviations and acronyms were usually provided (54%, 13/24). The descriptions used by the guideline development group to make decisions were not usually described (38%, 9/24). Moreover, the external review (29%, 7/24), funding sources (25%, 6/24), and roles of the funder (25%, 6/24) were not adequately described. The ICC scores and diverse reporting guidelines and consensuses are summarized in Table 3.

**TABLE 3:** Reporting rates (%) and ICC score of included guidelines and consensus.

Included guidelines	Basic information	Background	Evidence	Recommen -dations	Review and quality assurance	Funding and declaration and management of interests	Other information	Total reporting rates
Hochberg et al. (19)	75.0	56.3	0	42.9	25.0	0	8.3	36.4
Godel (26)	75.0	56.3	15.0	42.9	0	0	16.7	37.9
Sanders et al. (21)	75.0	46.9	0	46.4	25.0	25.0	0	37.1
Holick et al. (15)	75.0	78.1	25.0	64.3	37.5	50.0	25.0	57.6
Vidailhet et al. (24)	62.5	56.3	0	28.6	0	0	50.0	33.6
GNS (29)	50.0	43.8	0	28.6	0	0	0	24.3
Braegger et al. (18)	54.2	68.8	10.0	35.7	0	25.0	0	36.4
SAHM (16)	62.5	34.4	0	28.6	0	0	0	24.3
Paxton et al. (22)	70.8	71.9	20.0	53.6	50.0	25.0	0	47.9
GNS (30)	58.3	62.5	0	35.7	0	0	0	31.4
Płudowski et al. (32)	70.8	62.5	0	50.0	0	0	33.3	39.3
Golden et al. (14)	29.2	65.6	0	35.7	0	50.0	33.3	34.9
Munns et al. (11)	83.3	68.8	30.0	60.7	12.5	50.0	33.3	55.7
Grossman et al. (17)	66.7	53.1	0	32.1	0	0	0	30.0
CSOBMR (12)	75.0	56.3	0	35.7	0	0	0	32.9
Haq et al. (23)	66.7	68.8	0	39.3	0	0	16.7	36.4
Rusinska et al. (27)	91.7	93.8	40.0	60.7	0	50.0	33.3	64.4
Saggese et al. (28)	75.0	81.3	10.0	50.0	0	25.0	66.7	51.4
CHSCPMA (4)	54.2	50.0	0	28.6	0	0	0	26.4
Palacios et al. (31)	70.8	50.0	25.0	46.4	0	25.0	0	39.7
CHSCPMA (5)	54.2	68.8	0	35.7	0	0	0	32.1
Gupta et al. (20)	100.0	81.3	20.0	71.4	25.0	50.0	50.0	65.2
Bacchetta et al. (25)	83.3	75.0	80.0	71.4	25.0	37.5	58.3	69.7
PSCMA (13)	83.3	81.3	40.0	64.3	75.0	68.8	66.7	69.3
ICC (95%CI)	0.92 (0.89–0.94)	0.84 (0.80–0.88)	0.94 (0.92–0.96)	0.89 (0.85–0.92)	0.93 (0.88–0.96)	0.98 (0.97–0.99)	0.87 (0.80–0.92)	—
Mean (SD)	69.3 (14.9)	63.8 (14.1)	13.1 (19.6)	45.4 (14.0)	11.5 (19.8)	20.1 (22.9)	20.5 (23.7)	42.3 (14.3)

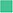
 100%∼80%; 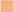 79%∼60%; 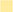 59%∼40%; 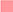 39%∼20%; 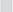 <20%.

ICC, intra-class correlation coefficient; SAHM, society for adolescent health and medicine; CSOBMR, Chinese society of osteoporosis and bone mineral research; CHSCPMA, child health society of Chinese preventive medicine association; GNS, German Nutrition Society; PSCMA, Pediatrics Society of Chinese Medical Association.

### Analysis of overall quality

The overall quality of the included guidelines and consensus was low, and the comprehensive methodological quality and reporting quality of evidence-based guidelines are higher than those of non-evidence-based guidelines and consensus. Only seven guidelines were recommended with modifications, and had an overall reporting rate of over 50%. The AGREE II score and RIGHT reporting rate summary analysis results are shown in Supplementary material 2.

### Analysis of recommendations

A total of three calcium supplementation guidelines and consensuses (4, 21, 30), 17 VD supplementation guidelines and consensuses (5, 12, 13, 15–18, 20, 22–24, 26–29, 31, 32), and four calcium and VD supplementation guidelines were included (11, 14, 19, 25). These sources recommended preventive or upper limit doses and serum 25(OH)D levels for healthy children of different ages; details on the preventive dose, upper limit dose, choice of VD2 or VD3 supplements, sunlight exposure suggestion, and serum 25(OH)D levels are shown in Supplementary materials 3, 4.

### Recommendations on calcium and vitamin D supplementation

The daily intake recommendations for calcium or VD vary considerably according to the guidelines and consensuses referenced. Regarding the preventive calcium dose, two guidelines (8%, 2/24) recommended calcium supplementations for children ranging from 400 to 1,150 mg per day.

Regarding preventive VD dose, most guidelines (50%, 12/24) recommended daily intake ranging from 400 to 600 IU/day. A total of five guidelines and consensuses recommended an upper limit dose ranging from 1,000 to 4,000 IU per day. An additional file shows this in more detail in Supplementary material 3.

### Choice of VD2 or VD3 supplements

Regarding the choice between VD2 and VD3 supplements, three guidelines and consensuses were considered both to be equivalent (12, 14, 15), while five suggested that VD3 has a better effect (13, 16, 20, 22, 24), and two guideline recommended that VD3 has a better effect with a single high dose than VD2 (11, 25). An additional file shows this in more detail in Supplementary material 3.

### Sunlight exposure and serum concentration of 25-hydroxyvitamin-D

Seventeen guidelines and consensuses (71%, 17/24) recommended obtaining VD from sunlight to maintain bone health. Meanwhile, 20 guidelines and consensuses (83%, 20/24) recommended serum concentration levels of 25(OH)D greater than 50 or 75 nmol/L (i.e., 20 or 30 ng/mL), and used words, such as “optimal,” “sufficient/sufficiency,” “adequate,” “at least,” and “obtain and maintain.” An additional file shows this in more detail in Supplementary materials 3, 4. The majority of guidelines and consensuses (71%, 17/24) did not recommend routine screening of serum 25(OH)D levels in healthy children, but did recommend the screening of children at risk of VD deficiency, such as those with a dark skin tone, exposure to winter and sunlight time, and dressing (e.g., covering skin for cultural reasons) and sun protection.

## Discussion

Calcium and VD are two essential nutrients for normal bone growth and maintenance. Calcium plays a crucial role in skeletal mineralization, supporting bone strength and muscle contraction. VD facilitates calcium absorption *via* its active hormonal form, 1,25-dihydroxycholecalciferol [1,25(OH)2D3], working together with the parathyroid hormone to maintain calcium homeostasis (33). Deficiencies in calcium and VD are associated with rickets, osteomalacia, and an increased risk of fractures and osteoporosis (34, 35). Furthermore, excessive supplementation can lead to adverse events, including cardiovascular diseases and kidney stones (36, 37). To date, the appropriate target levels of calcium, VD supplementation, and serum 25(OH)D concentrations remain controversial (38). A high-quality guideline should not only be developed in a process that meets rigorous methodological standards, but it should also be reported clearly and completely. This study summarized current guidelines and consensus recommendations for calcium and VD supplementation in healthy children, assessed the methodological and reporting quality of guidelines and consensus using the AGREE II and RIGHT checklist, and analyzed the AGREE II scores for each domain, the overall assessment, and the RIGHT reporting rates for each domain and total reporting rates.

### Guideline quality analysis

The AGREE II evaluation results suggest that the overall quality of the guidelines and consensuses in this field was low, of which the quality in terms of scope, purpose, clarity, and presentation was relatively good, and the quality of formulation was low in terms of rigor of development and applicability. The following issues still need to be addressed: (1) The guidelines and consensuses should include the participation of multidisciplinary teams, such as methodologists and health economists. (2) Patient values and dietary preferences in the developmental stage must be considered. (3) A comprehensive literature search strategy with clear inclusion and exclusion criteria should be reported in detail. Quality evaluation, classification of evidence, and the development process or method of forming recommendations should also be described. The quality of the evidence supporting the recommendation (e.g., high, moderate, low, or very low) and the strength of the recommendation (e.g., strong, moderate, or weak recommendation) should be described so that users or readers of the guideline can accurately understand and apply the guideline. When developing recommendations, it is necessary to consider not only the effectiveness, but also safety using supporting data. (4) Adequate declaration and management of conflicts of interest should be carried out. (5) Lastly, the quality evaluation tool of guidelines should include guideline register protocols to increase the transparency of the guideline development process.

Similarly, the RIGHT evaluation results suggest that the overall reporting quality of the guidelines and consensuses was low. The domain with the highest reporting rate was basic information, and that with the lowest reporting rates were evidence, review, and quality assurance. The following issues still need to be addressed: (1) The year of publication should be indicated in the title or subtitle of the guidelines for a clearer understanding of users. (2) The target population and guideline setting should be reported clearly and accurately, so that the guidelines can be better promoted and applied. All contributors who participate in the guidelines development and their roles should be described. (3) It is recommended that the key issues of the guidelines be provided in the population, intervention, comparisons, and outcomes format. (4) The description and specifications of the review and quality control procedures should be addressed. (5) Conflicts of interest and their management strategies should be fully reported to improve the credibility of the guidelines. (6) Finally, relevant reports on the accessibility of the guidelines, limitations, and suggestions for future research should be increased.

### Analysis of differences in recommendations

The guidelines and consensuses of different countries or regions differ greatly in recommendations regarding the necessity, dosage, and upper limit dose of calcium supplementation and VD in healthy children. The possible reasons underlying these differences are as follows: (1) Guidelines and consensuses regarding the definition of VD deficiency remain controversial, and test methods for serum 25(OH)D levels have not been standardized; (2) regions with different latitudes and altitudes differ in sunlight intensity, and there are differences in the basic VD status of healthy children, resulting in inconsistent recommended serum 25(OH)D thresholds (12, 25); (3) the dietary habits, race, skin color, sun protection measures (e.g., using sunscreen), air pollution, economy, and cultures of various countries are also possible factors that affect the recommendations (12, 39). Previous studies have shown that as altitude increases, VD synthesis by skin increases (40, 41). People with dark skin are associated with a higher risk for VD deficiency than those with light skin, due to less efficient VD synthesis by their skin. Furthermore, the incidence of VD deficiency might be higher in air-polluted areas than in pollution-free areas (40). (4) Differences in the methods of formulating guidelines (e.g., evidence-based versus non-evidence-based guidelines), sources of evidence, evidence quality classification, and methods of development by different institutions (e.g., GRADE approach or other methods) may also affect the recommendations. (5) The lack of dose-response trials of vitamin D in children and adolescents has also hindered the development of evidence-based dietary requirements for vitamin D in children (and therefore guidelines).

The adequacy of vitamin D in the body can be estimated by measuring the serum 25(OH)D concentration. Most guidelines and consensus recommend that the serum 25(OH)D concentration should be higher than 20 ng/mL (50 nmol/L). Studies (42, 43) have found that when high-dose VD is supplemented (such as 50,000 IU single dose), the absolute value of serum 25(OH)D increased by VD3 is significantly higher than that of VD2. The difference between the two types of vitamin D was not as pronounced when supplemented with small daily doses of vitamin D. The reason may be that the half-life of 25(OH)D3 is longer than that of 25(OH)D2, and the affinity of VD-binding protein for 25(OH)D3 is greater than that of 25(OH)D2, and the effect of promoting calcium absorption is better (44, 45). Therefore, in combination with the included guidelines and consensus and the results of relevant studies, the view that VD2 and VD3 are equivalent should be treated with caution.

### Strengths and limitations

To the best of our knowledge, this is the first review of the recommendations on calcium and VD supplementation in healthy children, based on the AGREE II and RIGHT evaluations. Previously, researchers have evaluated the quality of the guidelines on VD supplementation in children with VD deficiency (46, 47). However, our study focuses on preventing calcium and VD deficiency in healthy children, which has broader population needs and application bases.

Some limitations indicated are as follows: (1) As calcium or VD supplementation in healthy children is quite common in various countries, this study did not include some non-English guidelines, resulting in a language selection bias. (2) Some items of the AGREE II or RIGHT evaluations lacked clear criteria for judgment or were determined to be inapplicable. Therefore, we could not rule out the subjectivity of certain item evaluations, and the scoring scale needs to be further optimized and refined. (3) There may be contradictions in that the guideline qualities were low, but are widely used in clinical practice. This is because the AGREE II instrument can only be used to evaluate the guideline formation process rather than the actual validity of the guideline recommendations. To address the conflicting question of methodological quality and clinical validity, other evaluation tools should also be combined, such as AGREE-REX (48).

## Conclusion

Recommendations on calcium and VD in healthy children showed substantial variation in a set of 24 guidelines and consensuses across countries worldwide. Further research is needed to explore the reasons for these differences, providing scientific basis for the formulation of public health strategies for children. A team of clinicians, nutritionists, methodologists, health economists, etc., will develop localized recommendations based on the characteristics of the region. The methodological quality and reporting quality of the existing guidelines and consensuses in healthy children must be improved. Guideline developers need to strengthen the standardization of the guidelines to improve their quality and formulate trustworthy recommendations.

## Data availability statement

The original contributions presented in this study are included in the article/Supplementary material, further inquiries can be directed to the corresponding author.

## Author contributions

PZ and SZ conceived and designed the study. LH and PZ conducted the data screening and extraction. LH and XZ appraisers with experience in quality assessment of guidelines, scored each guideline using the AGREE II instrument and RIGHT statement and collected and analyzed the data. LH wrote the first draft of the manuscript. PZ, SZ, ST, and JH critically revised the manuscript in response to feedback. All authors have read and approved the final manuscript.

## Footnotes

1
http://www.guideline.gov

2
http://www.g-i-n.net/

3
http://www.nice.org.uk/

4
http://www.sign.ac.uk/

5
http://www.guidelines-registry.cn/

6
http://www.who.int/en/

7
https://www.aap.org

8
https://nutrition.org/

9
https://fensnutrition.org/

10
https://www.agreetrust.org/

## Supplementary material

Supplementary material 1 Sources searched and search strategies.

Supplementary material 2 Overall quality of included guidelines and consensuses (bubble chart).

Supplementary material 3 Recommendations and basis for included guidelines and consensuses.

Supplementary material 4 Definition of vitamin D status and recommended populations for screening.

## Abbreviations

AGREE II: Appraisal of Guidelines for Research and Evaluation II
RIGHT: Reporting Items for Practice Guidelines in Healthcare
ICC: intra-class correlation coefficient
VD: vitamin D
CI: confidence interval
25(OH)D: 25-hydroxyvitamin-D
1,25(OH)_2_D_3_: 1,25-dihydroxycholecalciferol.
