# Supplementary material for: Evaluation of the clinical practice guidelines and consensuses on calcium and vitamin D supplementation in healthy children using the Appraisal of Guidelines for Research and Evaluation II instrument and Reporting Items for Practice Guidelines in Healthcare statement
Source: Front Nutr. 2022 Sep 27;9:984423. doi: 10.3389/fnut.2022.984423 (PMC9551644; doi:10.3389/fnut.2022.984423)
Supplement: Supplementary material 1 — Sources searched and search strategies. [file Data_Sheet_1.docx]

# Supplementary Material 1. Sources searched and search strategies

| Source | Search strategy | Hits  retrieved |
| --- | --- | --- |
| PubMed | | 549 |
| #1 | Calcium [MeSH] |  |
| #2 | calcium, dietary [MeSH] |  |
| #3 | Calcium [text word] |  |
| #4 | dairy products [MeSH] |  |
| #5 | Dairy [text word] |  |
| #6 | Milk [text word] |  |
| #7 | dietary supplements [MeSH] |  |
| #8 | vitamin d [MeSH Terms] |  |
| #9 | vitamin D [Text word] |  |
| #10 | Ergocalciferols [MeSH Terms] |  |
| #11 | Ergocalciferol [Text word] |  |
| #12 | Cholecalciferol [MeSH Terms] |  |
| #13 | Cholecalciferol [Text word] |  |
| #14 | Hydroxycholecalciferols [MeSH Terms] |  |
| #15 | Hydroxycholecalciferol [Text word] |  |
| #16 | 25-Hydroxyvitamin D 2 [MeSH Terms] |  |
| #17 | 25-hydroxyvitamin D 2 [Text word] |  |
| #18 | Dihydrotachysterol [MeSH Terms] |  |
| #19 | Dihydrotachysterol [Text word] |  |
| #20 | Dihydroxyvitamin D [Text word] |  |
| #21 | Calcitriol [MeSH Terms] |  |
| #22 | Calcitriol [Text word] |  |
| #23 | Alfacalcidol [Supplementary Concept] |  |
| #24 | alfacalcidol [Text word] |  |
| #26 | Alphacalcidol [Text word] |  |
| #27 | guideline [publication type] |  |
| #28 | practice guideline [publication type] |  |
| #29 | recommendation [title/abstract] |  |
| #30 | standard [title/abstract] |  |
| #31 | guideline [title/abstract] |  |
| #32 | position statement [title/abstract] |  |
| #33 | position paper [title/abstract] |  |
| #34 | Consensus [MeSH] |  |

| #35 | Consensus [title/abstract] |  |
| --- | --- | --- |
| #36 | health planning guidelines [MeSH] |  |
| #37 | guideline [publication type] |  |
| #38 | practice guideline [publication type] |  |
| #39 | OR/1-7 |  |
| #40 | OR/8-26 |  |
| #41 | 39 OR 40 |  |
| #42 | OR/27-38 |  |
| #43 | 41 AND 42 |  |
| #44 | All child [Filter] |  |
| Embase | | 4371 |
| #1 | ‘calcium’/exp |  |
| #2 | ‘calcium intake’/exp |  |
| #3 | calcium |  |
| #4 | ‘dairy products’/exp |  |
| #5 | dairy |  |
| #6 | milk |  |
| #7 | ‘dietary supplements’/exp |  |
| #8 | ‘vitamin d’/exp |  |
| #9 | ‘vitamin D’ |  |
| #10 | ‘Ergocalciferol’/exp |  |
| #11 | Ergocalciferol |  |
| #12 | ‘Colecalciferol’/exp |  |
| #13 | Cholecalciferol |  |
| #14 | ‘Hydroxycholecalciferol’/exp |  |
| #15 | hydroxycholecalciferol |  |
| #16 | ‘25 hydroxyergocalciferol’/exp |  |
| #17 | ‘25 hydroxyergocalciferol’ |  |
| #18 | ‘Dihydrotachysterol’/exp |  |
| #19 | dihydrotachysterol |  |
| #20 | ‘Dihydroxyvitamin d’ |  |
| #21 | ‘calcitriol’/exp |  |
| #22 | calcitriol |  |
| #23 | ‘alfacalcidol’/exp |  |
| #24 | alfacalcidol |  |
| #26 | alphacalcidol |  |
| #27 | ‘guideline’/exp |  |

| #28 | guideline |  |
| --- | --- | --- |
| #29 | ‘practice guideline’/exp |  |
| #30 | recommendation |  |
| #31 | standard |  |
| #32 | ‘position statement’ |  |
| #33 | ‘position paper’ |  |
| #34 | ‘consensus’/exp |  |
| #35 | consensus |  |
| #36 | ‘health care planning’/exp |  |
| #37 | OR/1-7 |  |
| #38 | OR/8-26 |  |
| #39 | 37 OR 38 |  |
| #40 | OR/27-36 |  |
| #41 | 39 AND 40 |  |
| # 42 | Newborn [Filter] OR infant [Filter] OR child [Filter] OR preschool child  [Filter] OR school child [Filter] OR adolescent [Filter] |  |
| CNKI | | 1019 |
| #1 | SU= calcium OR SU= calcium supplements OR SU= calcium carbonate OR SU= calcium chloride OR SU= calcium acetate OR SU= calcium bicarbonate OR SU= calcium hydrogen phosphate OR SU= calcium citrate OR SU= calcium lactate OR SU= calcium gluconate OR SU= dairy  products OR SU= dietary calcium OR SU= milk |  |
| #2 | SU= Vitamin D OR SU= ergosterol OR SU= cholecalciferol OR SU=  hydroxycholecalciferol OR SU= dihydroergosterol OR SU= calcitriol OR SU= α calcidol |  |
| #3 | SU= child OR SU= pediatric OR SU= newborn OR SU= infant OR SU= toddler OR SU= preschool OR SU= school age OR SU= adolescence OR  SU= teenager |  |
| #4 | TI= guideline OR TI= consensus OR TI= standard OR TI= guidance |  |
| #5 | 1 OR 2 |  |
| #6 | AND 3-5 |  |
| WanFang | | 766 |
| #1 | SU: (calcium) OR SU: (calcium supplements) OR SU: (calcium carbonate) OR SU: (calcium chloride) OR SU: (calcium acetate) OR SU: (calcium bicarbonate) OR SU: (calcium hydrogen phosphate) OR SU: (calcium citrate) OR SU: (calcium lactate) OR SU: (calcium gluconate)  OR SU: (dairy products) OR SU: (dietary calcium) OR SU: (milk) |  |
| #2 | SU: (Vitamin D) OR SU: (ergosterol) OR SU: (cholecalciferol) OR SU: |  |

|  | (hydroxycholecalciferol) OR SU: (dihydroergosterol) OR SU: (calcitriol)  OR SU: ( α calcidol) |  |
| --- | --- | --- |
| #3 | SU: (child) OR SU: (pediatric) OR SU: (newborn) OR SU: (infant) OR SU: (toddler) OR SU: (preschool) OR SU: (school age) OR SU:  (adolescence) OR SU: (teenager) |  |
| #4 | TI: (guideline) OR TI: (consensus) OR TI: (standard) OR TI: (guidance) |  |
| #5 | 1 OR 2 |  |
| #6 | AND 3-5 |  |
| SinoMed | | 491 |
| #1 | "calcium" [Common fields] OR "calcium supplements" [Common fields] OR "calcium carbonate" [Common fields] OR "calcium chloride " [Common fields] OR "calcium acetate" [Common fields] OR "calcium bicarbonate" [Common fields] OR "calcium hydrogen phosphate" [Common fields] OR "calcium citrate" [Common fields] OR "calcium lactate" [Common fields] OR "calcium gluconate" [Common fields] OR "dairy products" [Common fields] OR "dietary calcium" [Common fields]  OR "milk" [Common fields] |  |
| #2 | "Vitamin D" [Common fields] OR "ergosterol" [Common fields] OR "cholecalciferol" [Common fields] OR "hydroxycholecalciferol" [Common fields] OR "dihydroergosterol" [Common fields] OR  "calcitriol" [Common fields] OR "α calcidol " [Common fields] |  |
| #3 | "child" [Common fields] OR "pediatric" [Common fields] OR "newborn" [Common fields] OR "infant" [Common fields] OR "toddler " [Common fields] OR "preschool" [Common fields] OR "school age " [Common fields] OR "adolescence" [Common fields] OR "teenager" [Common  fields] |  |
| #4 | "guideline" [TI] OR "consensus"[TI] OR "standard"[TI] OR  "guidance"[TI] |  |
| #5 | 1 OR 2 |  |
| #6 | AND 3-5 |  |
| AHRQ [(http://w](http://www.guideline.gov/))w[w.guideline.gov/)](http://www.guideline.gov/)) | | 79 |
| #1 | Calcium |  |
| #2 | vitamin D |  |
| GIN [(http://w](http://www.g-i-n.net/))w[w.g-i-n.net/)](http://www.g-i-n.net/)) | | 16 |
| #1 | Calcium |  |
| #2 | vitamin D |  |
| NICE [(http://w](http://www.nice.org.uk/))w[w.nice.org.uk/)](http://www.nice.org.uk/)) | | 245 |
| #1 | Calcium |  |

| #2 | vitamin D |  |
| --- | --- | --- |
| SIGN [(http://w](http://www.sign.ac.uk/))w[w.sign.ac.uk/)](http://www.sign.ac.uk/)) | | 0 |
| #1 | Calcium |  |
| #2 | vitamin D |  |
| WHO [(http://w](http://www.who.int/en/))w[w.who.int/en/)](http://www.who.int/en/)) | | 11 |
| #1 | Calcium |  |
| #2 | vitamin D |  |
| AAP (https://www.aap.org) | | 146 |
| #1 | Calcium |  |
| #2 | vitamin D |  |
| ASN (https://nutrition.org/) | | 50 |
| #1 | Calcium |  |
| #2 | vitamin D |  |
| FENS (https://fensnutrition.org/) | | 107 |
| #1 | Calcium |  |
| #2 | vitamin D |  |
| IPGRP [(http://w](http://www.guidelines-registry.cn/))w[w.guidelines-registry.cn/)](http://www.guidelines-registry.cn/)) | | 19 |
| #1 | Calcium |  |
| #2 | vitamin D |  |


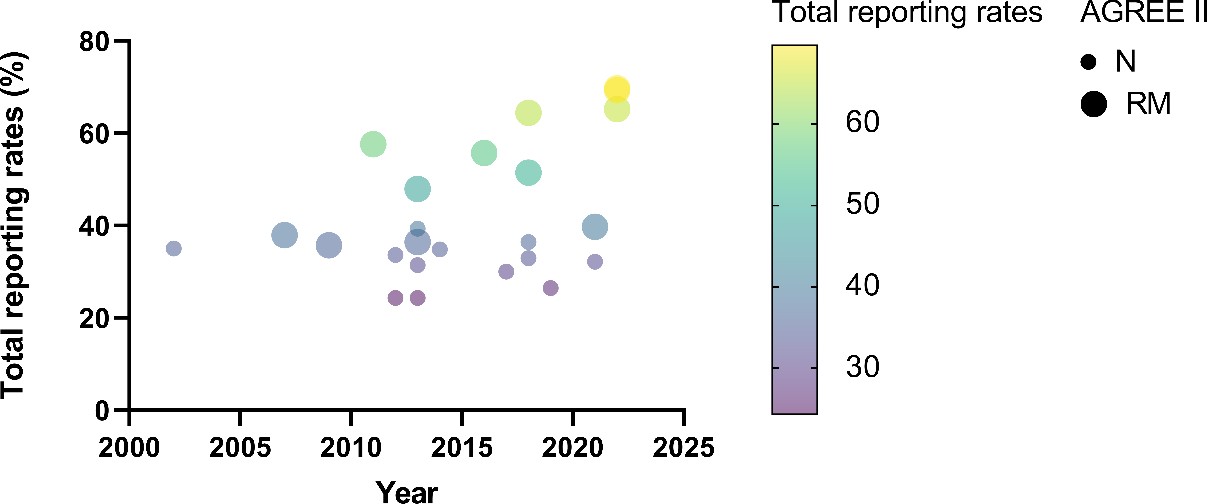


**Supplementary Material 2. Overall quality of included guidelines and consensus (bubble chart)**

AGREE II: Appraisal of Guidelines for Research and Evaluation Instrument II; RM: Recommended with modifications; N: Not Recommended

# Supplementary Material 3. Recommendations and basis for included guidelines and consensus

| Included guidelines | Recommendations | | | | Basis |
| --- | --- | --- | --- | --- | --- |
|  | Daily requirement | UL | VD2 or VD3  Supplements | Sunlight exposure |  |
| **Calcium** | | | | | |
| Sanders 2009 | Dietary calcium: Children and adolescents: 1000-1300 mg/d (800-  1050 mg/d)^b^ .  For children with very low calcium intakes, dietary modification or calcium supplementation is advisable | — | — | — | NHMRC/ literature |
| GNS 2013 | Infants  0 to <4 months ^a^: 220 mg/d;  4 to <12 months ^b^: 330 mg/d  Children  1 to <4 years: 600 mg/d;  4 to <7 years: 750 mg/d;  7 to <10 years: 900 mg/d;  10 to <13 years: 1100 mg/d; | — | — | — | IOM/literature |

|  | 13 to <15 years: 1200 mg/d;  Adolescents  15 to <19 years: 1200 mg/d;  ^a^ Estimated value for breastfed infants.  ^b^ Estimated value for the calcium intake from breast milk and solid  foods. |  |  |  |  |
| --- | --- | --- | --- | --- | --- |
| CHSCPMA 2019 | Dietary calcium^a^: 0-6m: 200 mg/d;  7-12m: 250 mg/d;  1-3y: 600 mg/d;  4-6y: 800 mg/d;  7-10y: 800 mg/d;  ＞11y: 1000 mg/d | — | — | — | CNS |
| **VD** | | | | | |
| Godel 2007 | VD supplementation: fullterm infant: 400 IU/d，with an increase to 800 IU/day from all sources between October and April north of the 55th  parallel (approximate | 1000 IU/d | — | Short periods (probably less than 15 min/day) | literature |

|  | latitude of Edmonton) and between the 40th and 55th parallel in individuals with risk factors for vitamin D deficiency other than  latitude alone |  |  |  |  |
| --- | --- | --- | --- | --- | --- |
| Holick 2011 | Dietary VD:  0-6m: 400-1000 IU/d;  6-12m: 400-1000 IU/d;  1-3y: 600-1000 IU/d;  4-8y: 600-1000 IU/d;  9-18y: 600-1000 IU/d | 0-6m: 2000IU/d;  6m-12m: 2000 IU/d;  1-3y: 4000 IU/d;  4-8y: 4000 IU/d;  9-18y: 4000 IU/d | Either VD2 or VD3 | — | IOM/literature |
| Vidailhet 2012 | VD supplementation:  ＜18m: 600-800 IU/d  （Formula feeding）, 1000-1200 IU/d（Breast feeding）;  18m-5y, 10-18y: 80,000 or  100,000 IU doses in winter, 1 in November, the other in February, which can be replaced by a single 200,000 IU dose when the risk of forgetting the 2nd dose seems high | — | VD3 | — | IOM |

| GNS 2012 | VD supplementation: Infants: 400 IU/d  Children: 800 IU/d  Adolescents: 800 IU/d | Infants: 1000 IU/d | — | regular sunlight exposure can prevent VD deficiency, but the safety is unknow | IOM/literature |
| --- | --- | --- | --- | --- | --- |
| Braegger 2013 | VD supplementation: 0-1y: 400 IU/d;  2-18y: 600 IU/d | 0-1y: 1000 IU/d;  1-10y: 2000 IU/d;  11-17y: 4000 IU/d | — | Regular sunlight exposure can prevent VD deficiency, but the safety is  unknow. | IOM/literature |
| SAHM 2013 | 12-18y: 600IU/d（400-  800 IU/d）, and at least 1,000 IU daily for adolescents who are at risk for VD defificiency or  insuffificiency | — | VD3 | Regular sunlight exposure can prevent VD deficiency, but the safety is unknow. | IOM/literature |
| Paxton 2013 | VD supplementation: 0（fullterm）-12m: 400IU/d;  1-18y: 400 IU/d or 150  000 IU at start of Autumn | — | VD3 | Regular sunlight exposure can prevent VD deficiency, but the safety is unknow. | IOM/literature |
| Płudowski 2013 | VD supplementation: 0-6m: 400IU/d;  6-12m: 400-600IU/d;  Children and adolescents (1–18 years, depending on body weight):  between September and  April: 600–1,000 IU/day; | neonates and infants: 1,000 IU/day;  1–10 years: 2,000 IU/day  11–18 years: 4,000 IU/day | — | Direct exposure to the sun is not recommended for infants younger than six months. Sun exposure is not recommended for people with skin type 1 (pale white skin, blue eyes, red hair) or with a history of sunburns or a history of skin cancer in the family.  For optimal effect, Central Europeans | European Food Safety Authority/literature |

|  | throughout the whole year: 600–1,000 IU/day (if sufficient skin syn thesis of Vitamin D is not  ensured in the summer) |  |  | should expose, without sunscreen, 18% of the body surface (i.e. uncovered forearms and partially exposed legs) to a half of one minimal erythemal dose  (MED, defined as slight reddening of skin 24 h post exposure) two or three  times per week |  |
| --- | --- | --- | --- | --- | --- |
| Grossman 2017 | without risk factors for vitamin D deficiency (Dietary VD):  1-18y: 600 IU/d  All infants should receive an oral supplementation of 400 IU/day of vitamin D. For children in high-risk groups, oral supplementation must be considered beyond 1 year of age. | infant: 1000 IU/d;  1-10y: 2000 IU/d;  11-17y: 4000 IU/d | — | Regular sunlight exposure can prevent VD deficiency, but the safety is unknow | ESPGHAN/  EFSA/literature |
| CSOBMR 2018 | VD supplementation: 0-12m: 400-1000 IU/d;  1-18y: 600-1000 IU/d | 0-6m: 1000 IU/d;  7-12m: 1500 IU/d;  1-3y: 2500 IU/d;  3-8y: 3000 IU/d;  ＞8y: 4000 IU/d | Either VD2 or VD3 | Spring, summer and autumn 11: 00 - 15: 00 Expose face and upper arms to sunlight for 5 to 30 minutes (depending on multiple factors), 3  times a week | IOM |

| Haq 2018 | VD supplementation^c^: 0-6m: 400 IU/d;  6-12m: 400-600 IU/d;  1-18y: 600-1000 IU/d | 0-6m: 1000IU/d;  6-12m: 1000IU/d;  1-10y: 2000IU/d;  11-18y: 4000IU/d | — | Regular sunlight exposure can prevent VD deficiency, but the safety is unknow | ES/IOM/ literature |
| --- | --- | --- | --- | --- | --- |
| Rusinska 2018 | VD supplementation: 0-6m: 400 IU/d;  6-12m: 400-600 IU/d;  1-10y:  May to September: supplementation is not necessary (if insolation  guidelines are met); Throughout a year: 600– 1000IU/day (If insolation guidelines are not fulfilled,  based on body weight and the dietary vitamin D intake);  11-18y:  May to September: supplementation is not necessary (if insolation  guidelines are met); Throughout a year: 800– 2000IU/day (If insolation  guidelines are not fulfilled, | neonates and infants: 1,000 IU/day;  1–10 years: 2,000 IU/day  11–18 years: 4,000 IU/day | — | In neonates, infants and children younger than 3 years of age, the direct exposure to sunlight without sunscreen is not recommended. In healthy children and adolescents sunbathing with uncovered forearms and legs for at least 15 min between 10.00 and  15.00 h, without sunscreen in the period from May to September | EVIDAS/literature |

|  | based on body weight and the dietary vitamin D  intake); |  |  |  |  |
| --- | --- | --- | --- | --- | --- |
| Saggese 2018 | <1y (full-term): 400 IU/d (without risk factors for vitamin D deficiency), up to 1000 IU/d (with risk factors for vitamin D deficiency)  1-18y: 600 IU/d (in presence of reduced sun exposure)-1000 IU/d (in presence of multiple risk factors for vitamin D deficiency).  In cases of poor compliance, supplementation with intermittent dosing (weekly or monthly doses for a cumulative monthly dose of 18000–30000 IU of vitamin D) can be considered, starting from  children aged 5-6 years | Infants: 1000 IU/d;  1-10y: 2000 IU/d;  11-17y: 4000 IU/d | — | — | ESPGHAN/EAP/EFSA |

|  | and particularly during  adolescent |  |  |  |  |
| --- | --- | --- | --- | --- | --- |
| Palacios 2021 | VD supplementation: 0-6m: 400 IU/d;  6-12m: 400-600 IU/d (the  dose is adapted to the type of diet);  1-11y: 600-1000 IU/d  (based on sun exposure guidelines);  12-18y: 800-2000 IU/d  (Supplementation is not necessary in healthy adolescents with good lifestyle, sun exposure and diet) | — | — | Sun bath with their forearms and legs uncovered for at least 15-20 min between 09:00 and 15:00 h, without sunscreen. | IOF/IOM |
| CHSCPMA 2021 | VD supplementation: Infant: 400-800 IU/d | — | — | Babies under 6 months of age should avoid direct sunlight; for high-risk  factors, take active sunlight exposure. | NRCGRPC/ IOM |
| Gupta 2022 | VD supplementation: Infant: 400 IU/d;  Childhood: 400 IU/d;  Adolescent: 600 IU/d | — | For daily intake， VD3 is only marginally better than VD2.  When intermittent bolus doses are used, | A daily sunlight exposure of 17-30 min in infants and 30-45 min in older children over 15-40% body surface area is recommended at least five times a week during noon (11AM- 3PM) for preventing VD deficiency  across. | IAP/literature |

|  |  |  | VD3 is more efficacious than VD2 | different regions and seasons (LOE 3.) |  |
| --- | --- | --- | --- | --- | --- |
| PSCMA 2022 | VD supplementation: 0-18y: 400 IU/d | — | VD3 is better | regular sunlight exposure can prevent VD deficiency, but the safety is  unknown. | IOM/GCRPMNR/literature |
| **Ca + VD** | | | | | |
| Hochberg 2002 | Calcium:  Term-first year: 400 mg/d Childhood: 800 mg/d  Adolescence: 1200 mg/d VD supplementation: Term-first year: 200-800 IU/d  Childhood: 0-400 IU/d^b^  Adolescence: 0-1000 IU/d^c^ ^b^ The higher dose is recommended for dark skin complexion and when sun exposure is limited.  ^c^ During childhood and adolescence, vitamin D requirement change with physical activity, geographical and cultural sun exposure and skin  complexion | — | — | 30 min/week clothed only in a diaper or 2 h/week fully clothed but no hat. Dark-skinned children will require longer exposures for infants. | literature |

|  |  |  |  |  |  |
| --- | --- | --- | --- | --- | --- |
| Golden 2014 | Dietary calcium^c^: 0-6m: 200 mg/d;  6-12m: 260 mg/d;  1-3y: 700 mg/d;  4-8y: 1000 mg/d;  9-13y: 1300 mg/d;  14-18y: 1300 mg/d Dietary VD^c^:  0-6m: 400 IU/d;  6-12m: 400 IU/d;  1-3y: 600 IU/d;  4-8y: 600 IU/d;  9-13y: 600 IU/d;  14-18y: 600 IU/d | calcium:  0-6m: 1000 mg/d;  6-12m: 1500 mg/d;  1-8y: 2500 mg/d;  9-18y: 3000 mg/d VD:  0-6m: 1000 IU/d;  6-12m: 1500 IU/d;  1-3y: 2500 IU/d;  4-8y: 3000 IU/d;  9-18y: 4000 IU/d | Both | — | IOM/literature |
| Munns 2016 | Dietary calcium: 0-6m: 200 mg/d;  6-12m: 260 mg/d Dietary VD and/or VD supplementation:  0-12m: 400 IU/d;  1-18y: at least 600 IU/d | — | For daily intake， equivalent.  When single large doses are used, VD3 is better. | regular sunlight exposure can prevent VD deficiency, but the safety is unknown. | IOM |
| Bacchetta 2022 | Calcium:  0-6m: 200 mg/d;  7-11m: 280 mg/d;  1-3y: 450 mg/d; | VD supplementation: 0-18y: 800 IU/d | daily supplementation: either VD2 or VD3 | regular sunlight exposure can prevent VD deficiency, but the safety is unknown. | EFSA/literature |

|  | 4-10y: 800 mg/d;  11-17y: 1150 mg/d; VD supplementation: 0-18y: 400 IU/d |  | intermittent supplementation: VD3 |  |  |
| --- | --- | --- | --- | --- | --- |

Note: IOM: International Osteoporosis Foundation; CNS: Chinese Nutrition Society; NHMRC: National Health and Medical Research Council; ESPGHAN: European Society for Pediatric Gastroenterology, Hepatology, and Nutrition; EFSA: European Food Safety Authority; ES: Endocrine Society; EAP: European Academy of Pediatrics; EFSA: European Food Safety Authority; IOF: International Osteoporosis Foundation ; NRCGRPC: the National Research Collaborative Group on Rickets Prevention and Control; m:months; y:years; UL: tolerable upper intake level; ^a^AI: Adequate Intake, the average observed daily level of intake by a population group of

apparently healthy people that is assumed to be adequate; ^b^EAR: estimated average requirement, meets the needs of 50% of the population; ^c^RDA: Recommended Dietary Allowance, the estimated intake capable of satisfying the needs of 97.5% of the population; SAHM：Society for Adolescent Health and Medicine；CSOBMR: Chinese Society of Osteoporosis and Bone Mineral Research; CHSCPMA: Child Health Society of Chinese Preventive Medicine Association; GNS: German Nutrition Society; PSCMA: Pediatrics Society of Chinese Medical Association; GCRPMNR: Global Consensus Recommendations on Prevention and Management of Nutritional

Rickets; EVIDAS: European Vitamin D Association; 1μg = 40 IU

# Supplementary Material 4. The definition of vitamin D status and the recommended population for screening

| Included  guidelines | Severe  deficiency | Deficiency | Insufficiency | Sufficiency/Adequacy | Children recommended for screening |
| --- | --- | --- | --- | --- | --- |
| **Calcium** | | | | | |
| Sanders 2009 | — | — | — | — | — |
| GNS 2013 | — | — | — | — | — |
| CHSCPMA 2019 | — | — | — | — | — |
| **VD** | | | | | |
| Godel 2007 | — | ＜10 ng/ml | 10-30 ng/ml | 30-90 ng/ml | — |
| Holick 2011 | — | ＜20 ng/ml | 21-29 ng/ml | ≥30 ng/ml | At risk of vitamin D deficiency |
| Vidailhet 2012 | — | ＜20 ng/ml | — | ≥20 ng/ml | At risk of vitamin D deficiency |
| GNS 2012 | — | — | — | ＞20 ng/ml | — |
| Braegger 2013 | ＜10 ng/ml | — | — | ≥20 ng/ml | — |
| SAHM 2013 | — | ＜20 ng/ml | 20-29 ng/ml | ≥30 ng/ml | At risk of vitamin D deficiency |
| Paxton 2013 | ＜5 ng/ml | 5-11 ng/ml | 12-19 ng/ml | ≥20 ng/ml | At risk of vitamin D deficiency |
| Płudowski 2013 |  | ＜20 ng/ml | 20-30 ng/ml | 30-50 ng/ml | At risk of vitamin D deficiency |
| Grossman 2017 | ＜10 ng/ml | — | — | ＞20 ng/ml | At risk of vitamin D deficiency |
| CSOBMR 2018 | ＜10 ng/ml | ＜20 ng/ml | 20-30 ng/ml | ＞30 ng/ml | At risk of vitamin D deficiency and need to  maintain a reasonable vitamin D nutritional status |
| Haq 2018 | — | ＜20 ng/ml | 21-29 ng/ml | ≥30 ng/ml | At risk of vitamin D deficiency |

| Rusinska 2018 | ＜10 ng/ml | 10-20 ng/ml | 20-30 ng/ml | 30-50 ng/ml | At risk of vitamin D deficiency |
| --- | --- | --- | --- | --- | --- |
| Saggese 2018 | ＜10 ng/ml | ＜20 ng/ml | 20-29 ng/ml | ≥30 ng/ml | At risk of vitamin D deficiency |
| Palacios 2021 | ＜10 ng/ml | 10-20 ng/ml | 20-29 ng/ml | 30-60 ng/ml | At risk of vitamin D deficiency |
| CHSCPMA 2021 | — | ＜12 ng/ml | 10-20 ng/ml | ＞20 ng/ml | — |
| Gupta 2022 | — | ＜12 ng/ml | 12-20 ng/ml | ＞20 ng/ml | At risk of vitamin D deficiency |
| PSCMA 2022 | — | ＜12 ng/ml | 12-20 ng/ml | 20-100 ng/ml | At risk of vitamin D deficiency |
| **Calcium + VD** | | | | | |
| Hochberg 2002 | ＜11 ng/ml | ＜25 ng/ml | 25-30 ng/ml | — | At risk of vitamin D deficiency |
| Golden 2014 | — | ＜20 ng/ml | — | ≥20 ng/ml | At risk of vitamin D deficiency |
| Munns 2016 | — | ＜12 ng/ml | 12-20 ng/ml | ＞20 ng/ml | At risk of vitamin D deficiency |
| Bacchetta 2022 | ＜10 ng/ml | ＜20 ng/ml | 20-29 ng/ml | 30-60 ng/ml | At risk of vitamin D deficiency |

Note: 1 ng/ml = 2.5 nmol/l; 1 nmol/l = 0.4 ng/ml; SAHM: Society for Adolescent Health and Medicine; CSOBMR: Chinese Society of Osteoporosis and Bone Mineral Research; CHSCPMA: Child Health Society of Chinese Preventive Medicine Association*;* GNS: German Nutrition Society; PSCMA: Pediatrics Society of Chinese Medical Association
